# Pharmacokinetic, Pharmacodynamic, and Clinical Outcomes of Vancomycin and β-Lactam Antibiotics in Adults With Augmented Renal Clearance: A Systematic Review

**DOI:** 10.7759/cureus.116657

**Published:** 2026-09-21

**Authors:** Mohammed Asiri, Sarah Alharbi, Mashail Alharbi, Ghaleb Alharbi

**Affiliations:** 1 Pharmacy Department, Prince Sultan Military Medical City, Riyadh, SAU; 2 Department of Pharmacy, King Abdullah Specialized Hospital, Ministry of National Guard Health Affairs, Buraydah, SAU; 3 Department of Clinical Pharmacy, College of Pharmacy, Shaqra University, Shaqra, SAU

**Keywords:** augmented renal clearance (arc), critical care, pharmacodynamics (pd), pharmacokinetic/pharmacodynamic target attainment (pk/pd), pharmacokinetics (pk), therapeutic drug monitoring (tdm), vancomycin, β-lactam antibiotics

## Abstract

Augmented renal clearance (ARC) is frequently seen in the critically ill population and may lead to faster clearance of antibiotics with renally dependent clearance. For β-lactam antibiotics and vancomycin, this may compromise drug exposure and pharmacokinetic/pharmacodynamic (PK/PD) target attainment, which is a clinically relevant risk for sub-therapeutic drug exposure and treatment failure. The aim of this study was to systematically review pharmacokinetic, pharmacodynamic, microbiological, and clinical outcomes of vancomycin and β-lactam antibiotics in adults with ARC. This systematic review was performed following Preferred Reporting Items for Systematic Reviews and Meta-Analyses (PRISMA) 2020 guidelines and was registered with the International Prospective Register of Systematic Reviews (PROSPERO) (CRD420261300771). Studies in which adults with ARC were treated with either vancomycin or β-lactams and had data on at least one of the pharmacokinetics, pharmacodynamics, microbiology, and/or clinical endpoints were identified in PubMed, Scopus, and Cochrane Library databases. Data synthesis is presented narratively following Synthesis Without Meta-analysis (SWiM) guidelines. There were 13 studies, all conducted mostly in intensive care units. Overall, in the included studies, the most frequent pharmacokinetic pattern for the studied antibiotics was reduced clearance and lower systemic exposure of antibiotics in patients with ARC. For vancomycin, in a retrospective cohort study, more than 60% of patients with ARC had their trough levels below 10 µg/mL. In another small randomized vancomycin trial, with once-every-eight-hours dosing, area under the concentration-time curve (AUC)/minimum inhibitory concentration (MIC) above 400 mg·h/L at MIC of 1 mg/L was reached in 82.14% of participants compared to 46.42% with once-every-twelve-hours dosing. In the case of β-lactams, improved PK/PD target attainment with prolonged and continuous infusion was seen in some studies. However, clinical and microbiological results were scarce. In general, ARC was linked with enhanced antibiotic clearance, decreased exposure, and inadequate PK/PD target attainment on standard dosing regimens. Optimization of dose regimens could help in improving target attainment; however, present evidence is not enough to show that there is an improvement in clinically important outcomes.

## Introduction and background

Augmented renal clearance (ARC) is a clinically important pharmacokinetic alteration characterized by enhanced renal elimination of drugs, most commonly operationalized as measured urine creatinine clearance (CrCl) >130 mL/min/1.73 m² in critical care research and prediction tools [1-4]. This threshold was selected a priori for the current review because it is the most frequently used definition in antibiotic pharmacokinetic (PK) studies and consensus-oriented reviews, but alternative thresholds have been reported, generally ranging from approximately 120 to 150 mL/min/1.73 m², with some earlier studies also using sex-specific absolute thresholds [2,4-5]. In fact, despite ARC representing an improved state of renal function, the increased rate of clearance of the antibiotics by the kidney can result in reduced exposure to the drugs. ARC is common rather than exceptional: a 2022 systematic review and meta-analysis estimated a pooled prevalence of 39% (95% CI: 34.9-43.3) across critically ill adults, with higher prevalence in neurocritical care populations, where pooled prevalence approached 74% [3]. ARC is most often described in younger patients, trauma populations, and those with neurologic injury, but it may also occur in non-ICU hospitalized adults [1,3-4,6].

Optimizing antimicrobial therapy in patients with serious infections remains difficult because drug exposure can vary substantially from one patient to another. This is especially relevant in critically ill patients and in those with altered renal function, where standard dosing may not reliably achieve pharmacokinetic/pharmacodynamic (PK/PD) targets. β-lactams and vancomycin are often prescribed in the treatment of serious infections among critically ill patients, hence the need for proper exposure to these drugs. For this reason, individualized dosing approaches are increasingly important in clinical practice [7-9].

Although advances in therapeutic drug monitoring (TDM) and model-informed precision dosing (MIPD) offer promising strategies [10-13], their routine clinical application remains limited by gaps in the evidence base. In the current literature, ARC is consistently associated with reduced antibiotic exposure and has been linked to subtherapeutic vancomycin concentrations and other PK/PD failures in some populations [3-4]. However, many studies rely on pharmacokinetic surrogate markers rather than patient-important outcomes. Furthermore, the current evidence is methodologically diverse with respect to ARC definition, assessment of CrCl using time-based versus estimated approaches, consideration of extracorporeal support and obesity/body size, presentation of co-therapies and dose adjustments, and the relationship between PK data and microbiological and clinical outcomes.

The clinical consequences of ARC are especially significant for antimicrobial agents predominantly eliminated via renal pathways, including β-lactam antibiotics and vancomycin [2,5,12]. For vancomycin, the 2020 American Society of Health-System Pharmacists (ASHP)/Infectious Diseases Society of America (IDSA)/Pediatric Infectious Diseases Society (PIDS)/Society of Infectious Diseases Pharmacists (SIDP) consensus guideline recommends area under the concentration-time curve (AUC)-guided monitoring with an individualized AUC/minimum inhibitory concentration (MIC) target of 400-600 mg·h/L (assuming broth microdilution MIC of 1 mg/L) rather than trough-only monitoring, and more recent international and Japanese consensus guidance similarly support Bayesian or model-informed AUC estimation where feasible [10-13]. For β-lactams, efficacy is primarily linked to the percentage of the dosing interval during which free drug concentrations exceed the minimum inhibitory concentration (fraction of the dosing interval/time (fT) > MIC). Because ARC increases renal drug elimination, subtherapeutic exposure is a recurring concern for both drug classes [2,14].

The changing strategy of antimicrobial management, especially in terms of vancomycin AUC-based monitoring and the use of more personalized and modeling-based dosing regimens, serves as a clinically meaningful context for an appraisal of the contemporary scientific evidence on antimicrobial exposure in adult patients with ARC. Thus, this systematic review examines contemporary studies published from 2020 onward to integrate pharmacokinetic, pharmacodynamic, microbiological, and clinical data related to the dosing and monitoring of current antimicrobial agents. Early literature will be used only for background purposes when needed. In particular, this systematic review examines vancomycin and β-lactam treatment in adult patients with ARC, concentrating on PK variations, PD target achievement, microbiological and clinical outcomes, dosing strategies, heterogeneity in the definition of ARC and renal function evaluation, and the quality of evidence overall.

## Review

Methods

Study Design and Protocol Registration

Prospectively, the review was registered in the International Prospective Register of Systematic Reviews (PROSPERO) (CRD420261300771). Following registration, the eligibility criteria were updated by deleting the mandatory comparator criterion to include single-arm as well as comparative trials that study the clinical, microbiological, and PK/PD parameters of antimicrobials in patients with ARC. This change in criteria was made since a significant number of studies on ARC involved pharmacokinetic studies and dosing without a comparator, which otherwise could have been excluded from being included in the systematic review. The systematic review adhered to the Preferred Reporting Items for Systematic Reviews and Meta-Analyses (PRISMA) 2020 statement.

Research Question

In adults with augmented renal clearance receiving vancomycin or β-lactam antibiotics, what are the pharmacokinetic, pharmacodynamic, microbiological, and clinical outcomes?

Eligibility Criteria

The eligibility criteria have been developed in accordance with the Population-Exposure-Outcome (PEO) framework. The PEO framework has been selected because the objectives of the review are to describe the results in ARC patient populations for different dosing regimens, rather than to compare a specific intervention to a comparator. This review was conducted as a systematic review using a narrative synthesis approach. Studies were eligible if they enrolled adult patients aged ≥18 years with ARC, investigated β-lactam antibiotics or vancomycin, and reported at least one clinical, microbiological, or PK/PD outcome. The primary outcomes of interest were clinical outcomes (treatment success, all-cause mortality, microbiological eradication, and adverse drug reactions); pharmacokinetic and pharmacodynamic target attainment outcomes were considered secondary but were anticipated to constitute the predominant evidence base given the published literature in this field. Both comparative and single-arm studies were eligible, reflecting the limited and heterogeneous nature of ARC research and the need to capture pharmacokinetic characterization studies that do not include a formal control group. Population pharmacokinetic modeling studies were eligible if conducted using observed patient data from ARC populations. Pure Monte Carlo simulation studies and in silico analyses without patient-derived data were excluded. Eligibility criteria are presented in Table 1.

**Table 1:** Eligibility Criteria (PEO Framework) PEO, Population-Exposure-Outcome; fT: fraction of the dosing interval/time; PK/PD, pharmacokinetic/pharmacodynamic; AUC, area under the concentration-time curve; MIC, minimum inhibitory concentration.

Component	Inclusion Criteria	Exclusion Criteria
Population (P)	Adult patients ≥18 years with augmented renal clearance (ARC) defined as creatinine clearance (CrCl) ≥130 mL/min, measured CrCl ≥130 mL/min/1.73 m², or a clearly defined ARC subgroup reported by the study authors	Pediatric-only populations (age <18 years)
Exposure (E)	Administration of beta-lactam antibiotics or vancomycin in ARC patients, including standard dosing, dose-adjustment strategies, extended infusion, continuous infusion, PK/PD-guided dosing, therapeutic drug monitoring (TDM), or model-informed precision dosing (MIPD)	Studies investigating antibiotics other than β-lactams or vancomycin
Outcomes (O)	At least one clinical, microbiological, or PK/PD outcome. Clinical: clinical cure, treatment success or failure, mortality, ICU/hospital length of stay, adverse drug reactions (e.g., nephrotoxicity), antimicrobial resistance. Microbiological: eradication, persistence, relapse. PK/PD: AUC, clearance, trough levels, AUC/MIC, fT > MIC, TDM results	Studies not reporting at least one relevant clinical, microbiological, or PK/PD outcome
Study Design	Randomized controlled trials, prospective cohort studies, retrospective cohort studies, observational studies, and single-arm studies reporting ARC outcomes. Single-arm designs were eligible given the limited and heterogeneous nature of ARC research	Case reports, small case series (n < 10), reviews, editorials, protocols, and conference abstracts
Study Type	Human studies evaluating antibiotic therapy in ARC populations, including population pharmacokinetic studies using observed patient data	Animal studies, in vitro studies, and pure pharmacokinetic simulation or Monte Carlo modelling studies without patient data

Search Strategy

The databases PubMed, Scopus, and the Cochrane Central Register of Controlled Trials (CENTRAL) were searched on February 9, 2026, through structured combinations of controlled vocabularies (MeSH terms) and free-text keywords for ARC, β-lactam antimicrobials, vancomycin, pharmacokinetics, pharmacodynamics, and dosing. The search was limited to articles written in the English language that dealt with adult human research studies published from January 1, 2020, to January 31, 2026. The year 2020 was prespecified for the review to concentrate the review on contemporary literature to be relevant to contemporary antimicrobial dosing and monitoring practices, especially after the trend of moving to AUC-based monitoring of vancomycin and model-informed dosing of medications.

ARC for this review was mainly characterized by a creatinine clearance of ≥130 mL/min/1.73 m², which was aligned with the most frequently employed operational threshold for ARC in antibiotic pharmacokinetics and critical care literature. Studies that employed ARC criteria closely similar or slightly different to the above, such as a creatinine clearance of >130 mL/min or criteria expressed without body surface area correction, were included in the review when the researchers clearly stated that their subjects had ARC [1-4]. The reference list of all included studies was manually checked on the same date. No trial registries were searched. No search was done on EMBASE and Web of Science due to logistical and access reasons preset within the scope of the review. Lack of inclusion of EMBASE is noted as a crucial limitation, as pharmacokinetics studies, conference presentations, or other evidence uniquely present in EMBASE might be missed out. Full search strings, exactly as executed, are presented in Appendix 1.

Study Selection

Two reviewers independently screened all records against eligibility criteria using a piloted screening form; full-text articles were subsequently assessed for eligibility. Both reviewers completed a calibration exercise on a random sample of 20 records prior to independent screening to ensure consistent application of eligibility criteria. The discrepancies at both levels of screening were settled through discussion and consensus; a third reviewer was also available to adjudicate if necessary, though not required in this case. Inter-rater agreement was not formally assessed using, for example, Cohen's kappa; this is a limitation of our methodology.

Data Extraction

Two reviewers independently extracted data using a standardized extraction form that was piloted on two included studies before full extraction, with minor revisions made before commencing. Extracted variables included study characteristics, population characteristics (sample size, age, sex, ARC definition, and renal function assessment method), intervention characteristics (antibiotic agent, dose, infusion strategy, and TDM use), and all reported outcomes. Where reported, we also extracted factors expected to contribute to heterogeneity in antibiotic exposure or target attainment, including timed versus estimated creatinine clearance methodology, body size metrics, extracorporeal support, concurrent nephroactive or dose-altering co-therapies, and microbiological assumptions such as MIC source. Outcomes were prioritized in the following hierarchy: clinical outcomes (treatment success, mortality, length of stay, adverse drug reactions), microbiological outcomes (eradication, persistence), pharmacodynamic target attainment (AUC/MIC, fT > MIC, probability of target attainment), and pharmacokinetic parameters (clearance, AUC, trough concentrations). Where multiple publications reported data from the same population, the most comprehensive dataset was used, and the decision was documented in the extraction form. Disagreements between reviewers were resolved by discussion; no unresolved disagreements required third-party adjudication. Study authors were not contacted for additional data. The extraction sheet for all studies is provided in Appendix 1.

Two studies had the same first author, institution (First Affiliated Hospital of China Medical University, China), drug focus (vancomycin), and overlapping recruitment periods: Chu et al. [18] from May 2013 to October 2016 and Chu et al. [19] from July 2013 to December 2015. Although the studies addressed different analytic questions, partial patient overlap cannot be excluded. Both studies were retained in the qualitative synthesis; however, they were not combined in participant-count summaries within a single vancomycin outcome domain.

Risk of Bias Assessment

The risk of bias was independently assessed by two reviewers. In the case of randomized trials, the Cochrane Risk of Bias 2 (RoB 2) instrument [20] was utilized at the outcome level when the corresponding questions for the specific outcome could be appropriately rated, and there was a preference to use PK/PD target attainment, clinical cure, all-cause mortality, and adverse drug reaction outcomes, where available. The Risk Of Bias In Non-randomized Studies of Interventions (ROBINS-I) instrument [21] was strictly utilized only in non-randomized comparative/interventional studies for which group-level comparative information was available and when its domains were methodologically relevant [16]. Non-comparative single-arm PK/PD studies, population pharmacokinetic model-based studies, and dosing modeling studies without appropriate comparative intervention framework were not given an overall ROBINS-I rating. However, their methodological weaknesses were rated descriptively using the above criteria. Inter-rater agreement statistics in numeric form were not preplanned for calculation during the original review process and thus cannot be reported as kappa statistics; any discrepancies were settled through discussion, and this is noted as a limitation. The risk of bias assessment results were taken into consideration while analyzing individual studies’ findings and weighing up their evidentiary value in the narrative analysis.

Data Synthesis and Certainty of Evidence

Considering the predicted heterogeneity between the included studies in relation to study design, definition of ARC, type of antibiotics used, population characteristics, estimation of renal function, and outcome reporting, meta-analysis was deemed inappropriate. Narrative synthesis was conducted using Synthesis Without Meta-analysis (SWiM) guidelines. Heterogeneity was predicted not only based on the type of antibiotic and comparison drug but also because of differences between timed CrCl and estimated CrCl, ICU patient population, body size, extracorporeal support, and other concomitant therapies. In order to address heterogeneity, studies were classified in the first instance according to type of antibiotic (vancomycin versus β-lactams), followed by outcome domains, and then by study design and comparison group characteristics. Consideration of risk of bias judgments was included in the synthesis and conservative Grading of Recommendations Assessment, Development and Evaluation (GRADE) downgrading where appropriate.

No pooled measures of effect were estimated. The narrative synthesis described (1) pharmacokinetic outcomes, as the difference in clearance, trough concentrations, and AUC values between ARC and non-ARC groups and/or different approaches to dosing; (2) pharmacodynamic outcomes, as the proportion of patients with predefined PK/PD target achievement (AUC/MIC > 400 mg·h/L for vancomycin; fT > MIC as stated in individual studies for β-lactams); and (3) clinical outcomes, as rates/proportions of treatment success, mortality, and ADRs as reported in individual studies. The certainty of evidence was evaluated using the GRADE approach. The outcome-level risk of bias assessment results were preferred when available; otherwise, study-level risk of bias was used cautiously for the corresponding outcome domain, being explicitly accounted for in the GRADE evaluation process. The initial certainty of evidence was high for RCTs and low for observational studies, with possible downgrading for risk of bias, inconsistency, indirectness, and imprecision and upgrading due to large effect size when appropriate. The publication bias could not be formally tested due to the low number of heterogeneous studies per outcome domain. The results of the GRADE assessments are presented in Appendix 1.

Results

Study Selection and Characteristics

Thirteen studies met the eligibility criteria and were included in the final synthesis (Figure 1). Figure 1 shows a single-stage reduction from 369 identified records to 13 included studies after removal of 15 duplicates and exclusion of 325 records at title/abstract screening and 16 full-text articles. Included studies were published from 2020 onward and were conducted predominantly in intensive care or other inpatient settings. By country of primary institution, five studies were conducted in China, two in Iran, and one each in Italy, Japan, Saudi Arabia, and the United States. In addition, one study was a multinational phase 3 trial across 29 countries, and one was a multicenter collaboration across the United States and Europe. Study designs comprised three randomized controlled trials, two prospective observational studies, six retrospective observational studies, one population pharmacokinetic modeling study using observed patient data, and one prospective phase 1 pharmacokinetic study. Table 2 summarizes the study-level mix, which was dominated by ICU-based cohorts and vancomycin or meropenem evaluations.

**Figure 1:**
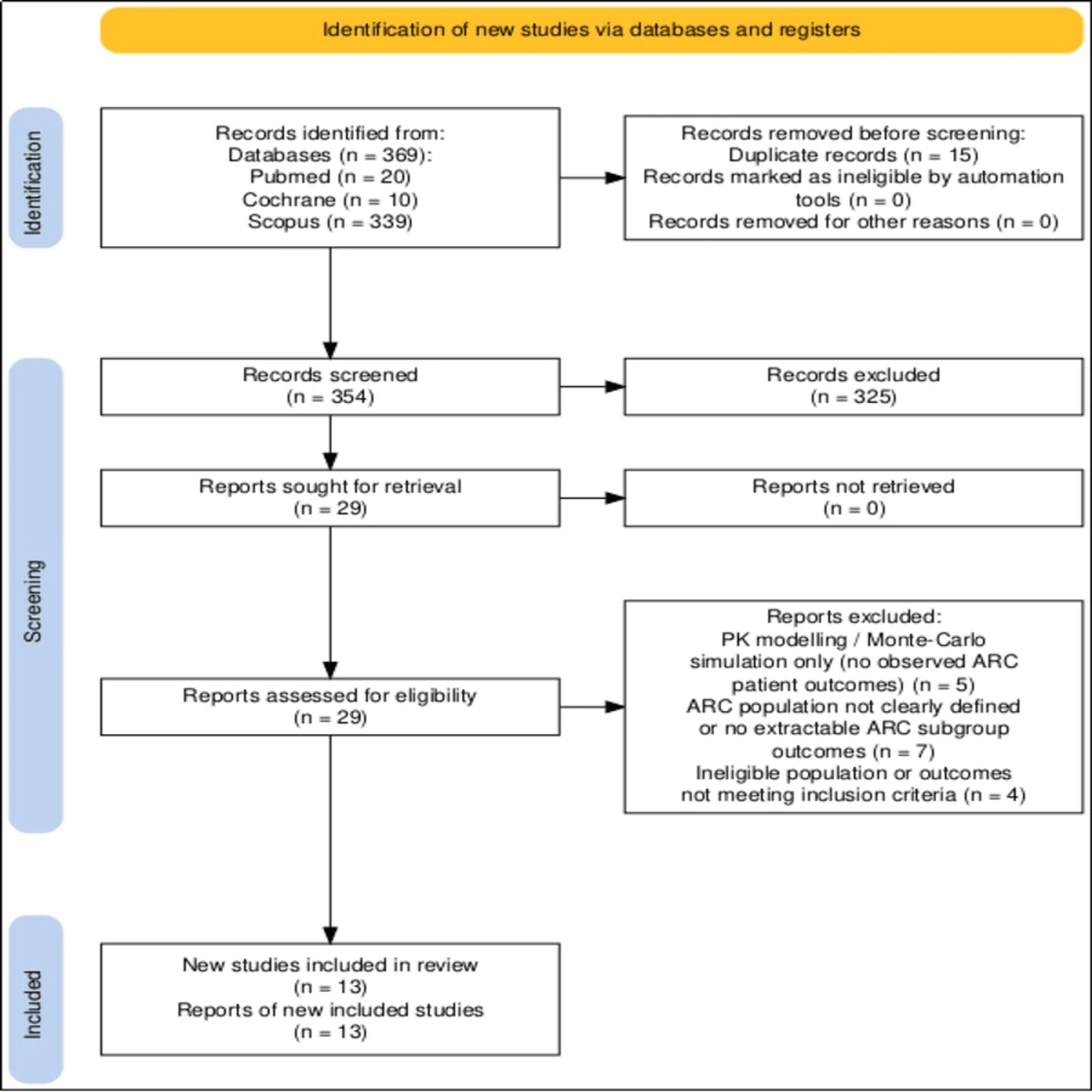
PRISMA 2020 Flow Diagram of Study Selection ARC, augmented renal clearance; PRISMA, Preferred Reporting Items for Systematic Reviews and Meta-Analyses; RCT, randomized controlled trial; PK, pharmacokinetic. Note: No additional records were found through hand-searching of the reference list. A summary of full-text exclusion category counts is presented in this figure.

**Table 2:** Characteristics of Included Studies ARC, augmented renal clearance; CrCl, creatinine clearance; ICU, intensive care unit; HAP/HABP, hospital-acquired (bacterial) pneumonia; VABP, ventilator-associated bacterial pneumonia; PLA: pharmacological analysis; TDM, therapeutic drug monitoring; PK, pharmacokinetic; RCT, randomized controlled trial; VAP, ventilator-associated pneumonia; popPK, population pharmacokinetic modelling; NONMEM, nonlinear mixed-effects modelling software. *Study period not reported in the original publication. †Very small sample size (n < 15); findings should be interpreted with caution given extremely limited statistical precision. ††Potential data overlap with Chu et al. [18]; both studies conducted at the First Affiliated Hospital of China Medical University with overlapping collection periods (July 2013-December 2015); see Methods and Limitations.

Author	Country	Journal	Design	Study Period	Setting	Population
Chu et al. [18]	China	Eur J Hosp Pharm	Retrospective observational	May 2013-Oct 2016	Inpatient, First Affiliated Hospital, China Medical University	Hospitalized patients with vancomycin TDM data; n = 292 (315 datasets)
Shorr et al. [22]	Multinational (29 countries)	Crit Care	RCT phase 3, post hoc ARC subgroup	Jan 2015-Apr 2018	ICU, ventilated patients	HABP/VABP patients; n = 726 total, 209 ARC
Sahraei et al. [23]	Iran	Front Pharmacol	RCT	Apr 2021-Jun 2022	ICU, Loghman Hakim Hospital	ICU patients with ARC receiving vancomycin; n = 56
Razzazzadeh et al. [24]	Iran	Eur J Clin Pharmacol	Single-center RCT	Oct 2019-Mar 2021	ICU, ventilated patients	VAP patients with ARC; n = 45
He et al. [25]	China	Transl Androl Urol	Retrospective observational	Jan 2013-Nov 2018	ICU, tertiary hospital	Critically ill receiving vancomycin; n = 280 (139 ARC)
Troisi et al. [26]	Italy	Pharmaceutics	Retrospective observational (prospective timed urine CrCl measurements applied within retrospective cohort)	Dec 2020-Jan 2022	Post-transplant ICU, Bologna	Critically ill adults, continuous meropenem; n = 46
Fratoni et al. [27]	USA	J Antimicrob Chemother	Single-center observational PK	Not reported*	ICU (medical, surgical, neurosurgical)	Critically ill with confirmed ARC; n = 8†
Mikami et al. [28]	Japan	J Infect Chemother	Retrospective observational	Apr 2014-Jul 2020	Emergency Dept, Hokkaido University	Critically ill receiving IV vancomycin; n = 65
Alsultan et al. [29]	Saudi Arabia	Saudi Pharm J	Prospective observational	Jul 2019-Mar 2020	Medical/Surgical ICU	Critically ill receiving meropenem; n = 43
Tang et al. [30]	China	Front Pharmacol	Retrospective observational	Jul 2016-Jun 2020	Maternal ICU, Suzhou	Critically ill obstetric patients; n = 427 (201 ARC)
Nicolau et al. [17]	USA/Europe (multicenter)	Int J Antimicrob Agents	Phase 1 prospective PK	Not reported*	ICU	Critically ill with confirmed ARC; n = 11†
Chen et al. [31]	China	J Int Med Res	Retrospective observational	Jan 2019-Jun 2019	Neurosurgery, PLA General Hospital	Post-neurosurgery receiving vancomycin TDM; n = 104
Chu et al. [19]††	China	J Infect Public Health	Retrospective + popPK modelling (NONMEM)	Jul 2013-Dec 2015††	First Affiliated Hospital, China Medical University	Hospitalized patients with ARC receiving vancomycin; n = 95

Population Characteristics and ARC Definitions

Sample sizes varied considerably, ranging from 8 to 726 participants. In studies with mixed ARC and non-ARC populations, the proportion of patients identified as having ARC ranged from approximately 25% to 64% of the study population (Table 3). ARC was most commonly defined as creatinine clearance exceeding 130 mL/min or 130 mL/min/1.73 m². Methods used to assess renal function were not uniform across studies, encompassing timed urine creatinine clearance (8-hour or 24-hour collections), Cockcroft-Gault estimates, and Chronic Kidney Disease Epidemiology Collaboration (CKD-EPI) estimated glomerular filtration rate (eGFR) formulae. ARC patients were predominantly younger to middle-aged adults (median or mean age ranging from approximately 33 to 50 years across cohorts). Most studies enrolled predominantly male patients, with the exception of one obstetric cohort comprising exclusively female patients. Table 4 summarizes sample characteristics and ARC definitions. Notably, the ARC threshold of 130 mL/min was relatively consistent across studies, whereas the method used to measure or estimate renal function varied considerably, with direct implications for patient classification accuracy and cross-study comparability and antibiotic therapy and dosing strategies (Table 4).

**Table 3:** Sample Characteristics and ARC Definitions ARC, augmented renal clearance; CrCl/CLcr/CCr/eCrCl, creatinine clearance; eGFR, estimated glomerular filtration rate; CKD-EPI, Chronic Kidney Disease Epidemiology Collaboration; IQR, interquartile range; SD, standard deviation; NR, not reported; M, male; F, female; BD, twice daily; TDS, three times daily. †Very small sample (n < 15) precision severely limited. ††Potential patient-level overlap with Chu et al. [18].

Author	Total n	ARC n	Comparator	ARC Definition	CrCl Method	Age (ARC)	Sex (ARC)
Chu et al. [18]	292	186	Non-ARC (CrCl <130)	CrCl > 130 mL/min	Cockcroft-Gault	Median 45 yrs (IQR: 33-57)	134 M/52 F
Sahraei et al. [23]	56	56 (all ARC)	None	Urinary CrCl > 130 mL/min	8-h urine collection	BD: 44 yrs; TDS: 43 yrs	BD: 60% male; TDS: 57% male
Shorr et al. [22]	726	209	Non-ARC subgroup	CrCl > 130 mL/min	Cockcroft-Gault	Not reported	Majority male (exact NR)
Razzazzadeh et al. [24]	45	45 (all ARC)	None	Measured CrCl ≥ 130 mL/min/1.73 m²	8-h urine collection	Not reported	Not reported
Troisi et al. [26]	46	12 (26.1%)	Non-ARC	Measured CrCl ≥ 130 mL/min/1.73 m²	24-h urine collection	NR for ARC subgroup (overall 58.5 yrs)	NR for ARC subgroup (overall 76.1% male)
Fratoni et al. [27]†	8	8 (all ARC)	None	CrCl ≥ 130 mL/min	8-h urine + Cockcroft-Gault screening	Mean 37 ± 14 yrs	62.5% male
Mikami et al. [28]	65	22	Non-ARC	CrCl > 130 mL/min on day 1	Daily 8-h urine collection	Median ~48-50 yrs	17 M/5 F
Alsultan et al. [29]	43	18 (42%)	Non-ARC	CrCl > 130 mL/min	Cockcroft-Gault	Mean 49 yrs	55% male
Tang et al. [30]	427	201 (47.1%)	Non-ARC	eGFR > 130 mL/min/1.73 m²	CKD-EPI equation	Not reported	Female only (obstetric)
Nicolau et al. [17]†	11	11 (all ARC)	None	Directly measured CrCl ≥ 130 mL/min	8-h urine collection	Mean 37 ± 9.6 yrs	54.5% male
He et al. [25]	280	139	Non-ARC	eCrCl > 130 mL/min	Cockcroft-Gault	40.0 ± 11 yrs	90 M/49 F
Chen et al. [31]	104	26	Non-ARC	CrCl > 130 mL/min/1.73 m²	Cockcroft-Gault	Median 33 yrs (IQR: 26-46)	18 M/8 F
Chu et al. [19]††	95	95 (all ARC)	None	CrCl ≥ 130 mL/min	Cockcroft-Gault	Median 45 yrs (IQR: 30-57)	71 M/24 F (74.7% male)

**Table 4:** Antibiotic Therapy and Dosing Strategies ARC, augmented renal clearance; IV, intravenous; q6h/q8h/q12h, every 6/8/12 hours; BD, twice daily; TDS, three times daily; TDM, therapeutic drug monitoring; eGFR, estimated glomerular filtration rate; HABP, hospital-acquired bacterial pneumonia; VABP, ventilator-associated bacterial pneumonia; VAP, ventilator-associated pneumonia; UTI, urinary tract infection; BSI, bloodstream infection; HAP, hospital-acquired pneumonia; C/T, ceftolozane/tazobactam. †Very small sample size (n < 15); findings should be interpreted with caution given extremely limited statistical precision. ††Potential data overlap with Chu et al. [18]; both studies were conducted at the First Affiliated Hospital of China Medical University with overlapping collection periods; see Methods and Limitations.

Author	Infection Type	Antibiotic	Class	Dose Strategy	Infusion	Dose Adjustment	TDM
Chu et al. [18]	Gram-positive infections	Vancomycin	Glycopeptide	1,000 mg q12h	Intermittent	None reported	Yes
Sahraei et al. [23]	Meningitis, pneumonia, septic shock	Vancomycin	Glycopeptide	Loading 20 mg/kg; maintenance 15 mg/kg q12h (BD) or q8h (TDS)	Intermittent	Not reported	Yes
Shorr et al. [22]	HABP/VABP	Ceftolozane/tazobactam; Meropenem	β-lactam; Carbapenem	C/T 3 g q8h; Meropenem 1 g q8h	1-h IV infusion	No ARC-specific adjustment	No
Razzazzadeh et al. [24]	VAP	Meropenem	Carbapenem	1 g q8h (3 h); 2 g q8h (3 h); 1 g q8h (6 h) 3 arms	Prolonged infusion	Yes dose and duration modified	No
Troisi et al. [26]	HAP; intra-abdominal	Meropenem	Carbapenem	Loading 2 g over 2 h; TDM-optimized maintenance	Continuous infusion (24 h)	Yes TDM-guided	Yes
Fratoni et al. [27]†	Documented/presumed infection	Imipenem/cilastatin/relebactam	Carbapenem + inhibitor	Single dose 1.25 g; simulations for 1.25 g q6h	30-min infusion	Not reported (simulation only)	No
Mikami et al. [28]	Not specified	Vancomycin	Glycopeptide	Physician-determined until first TDM	Not reported	Adjusted at first TDM	Yes
Alsultan et al. [29]	Pneumonia, intra-abdominal, UTI, BSI	Meropenem	Carbapenem	0.5-2 g adjusted per eGFR	Intermittent bolus	Yes eGFR-guided	Yes
Tang et al. [30]	Severe infection, obstetric ICU	Vancomycin	Glycopeptide	Not reported	Not reported	Not reported	Yes
Nicolau et al. [17]†	Proven/suspected infection	Ceftolozane/tazobactam	β-lactam	Single dose 3 g	60-min infusion	Not applicable (phase 1)	No
He et al. [25]	Intra-abdominal, sepsis, HAP	Vancomycin	Glycopeptide	15 mg/kg IV q12h	Intermittent (2 h)	Bayesian-guided TDM adjustment	Yes
Chen et al. [31]	Post-neurosurgery infections	Vancomycin	Glycopeptide	~1.28 g average (ARC group)	Not reported	Individualized	Yes
Chu et al. [19]††	Gram-positive infections	Vancomycin	Glycopeptide	Intermittent q6h-q12h; 1,000-4,000 mg/day	Intermittent IV	Not reported	Yes

Risk of Bias

Assessment of risk of bias in randomized and non-randomized comparative studies is shown in Figures 2, 3, respectively. The methodological quality of studies included varied with regard to missing data and confounding issues. These differences were taken into account while interpreting the results in narrative synthesis. In particular, the risk of bias assessment for Tang et al. [30] was re-evaluated, paying special attention to missing outcome data. Alsultan et al. [29] were identified as having serious risks of bias regarding confounding due to inadequate control of possible patient-, disease-, and treatment-related confounding factors. ROBINS-I was formally applied to non-randomized comparative/interventional studies with explicit group-level comparisons for which the tool was methodologically applicable. Single-arm PK/PD studies, population pharmacokinetic modeling studies, and non-comparative dosing/modeling studies are retained in the figure for completeness; however, they were not assigned an overall ROBINS-I judgment. For these studies, the displayed information should be interpreted as a descriptive assessment of relevant methodological limitations rather than a formal overall ROBINS-I classification.

**Figure 2:**
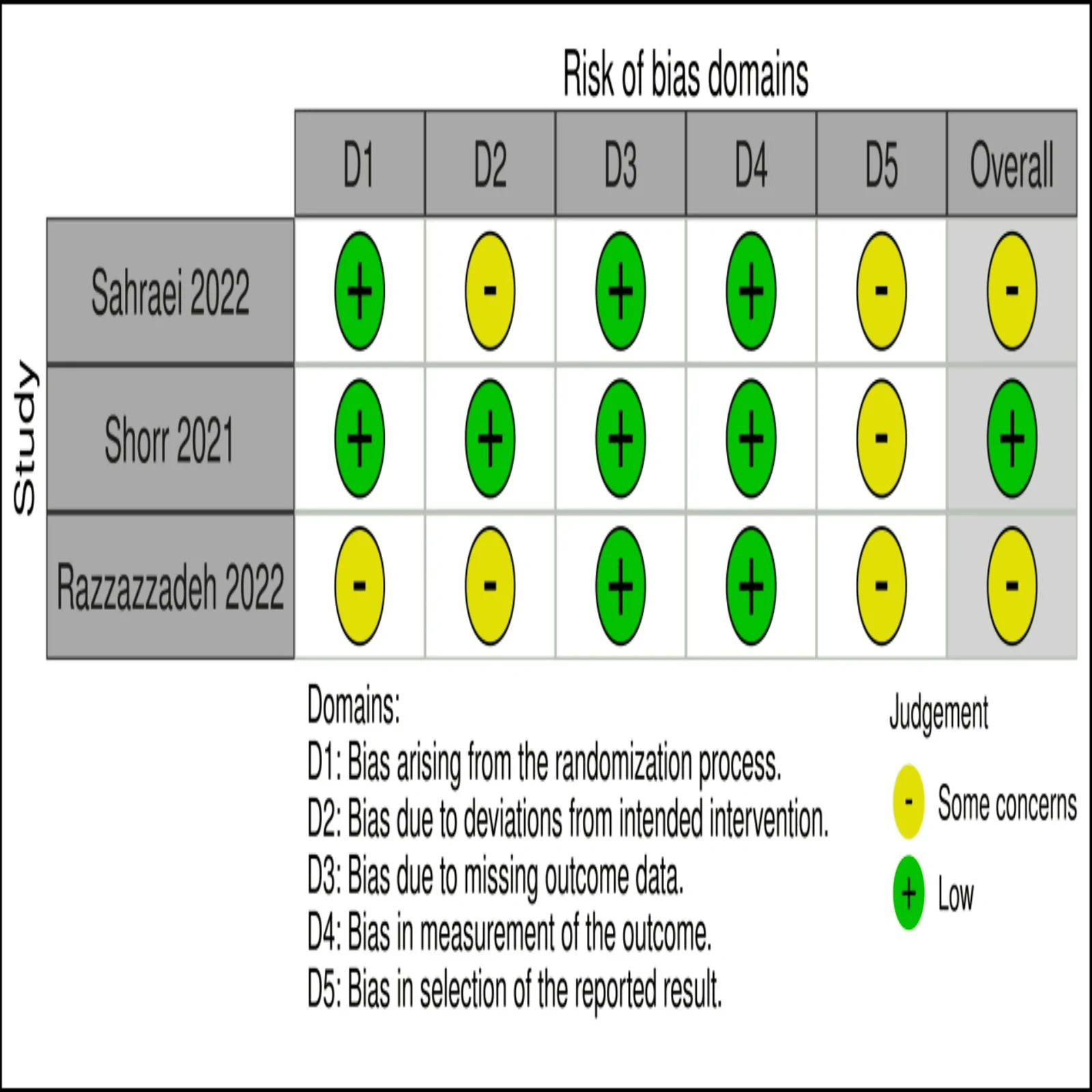
Risk of Bias Assessment of Randomized Controlled Trials Cochrane RoB 2 Tool Studies assessed were Shorr et al. [22], Sahraei et al. [23], and Razzazzadeh et al. [24]. The Cochrane Risk of Bias 2 (RoB 2) tool was used. Green (+) indicates low risk of bias, whereas yellow (-) indicates some concerns.

**Figure 3:**
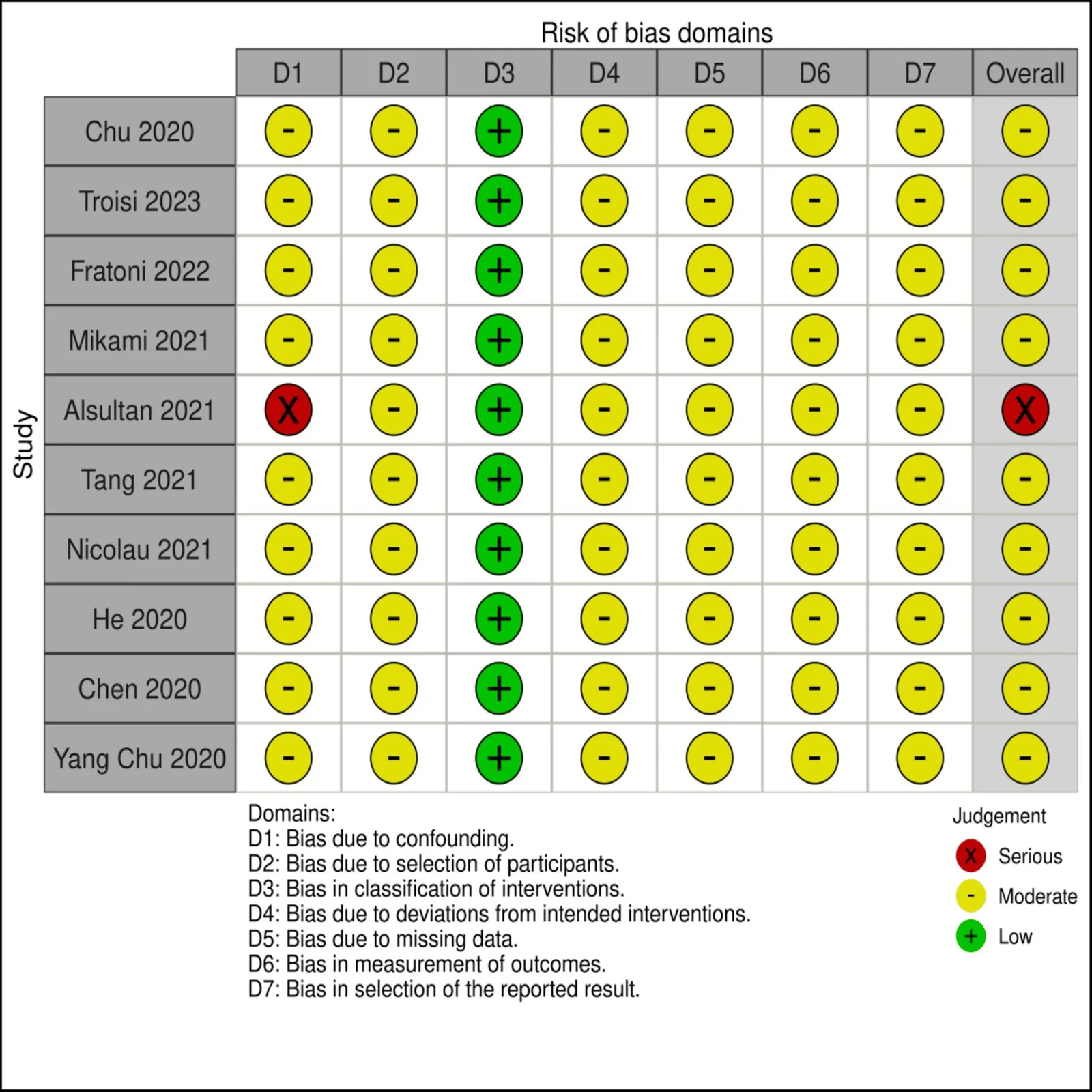
Risk of Bias Assessment of Non-Randomized Studies ROBINS-I Tool ROBINS-I: Risk Of Bias In Non-randomized Studies of Interventions. Studies assessed were Chu et al. [18], He et al. [25], Troisi et al. [26], Fratoni et al. [27], Mikami et al. [28], Alsultan et al. [29], Tang et al. [30], Nicolau et al. [17], Chen et al. [31], and Chu et al. [19]. The ROBINS-I tool was used. Green (+) indicates low risk, yellow (-) indicates moderate risk, and red (×) indicates serious risk of bias.

Pharmacokinetic Outcomes

Table 5 shows a consistent pharmacokinetic trend across 13 studies: ARC was associated with higher antibiotic clearance and lower systemic exposure, most clearly reflected by lower vancomycin troughs and AUC values and by higher β-lactam clearance. In vancomycin-treated cohorts, ARC patients consistently had lower trough concentrations and/or lower AUC exposure than non-ARC comparators. In Chu et al. [18], 186 ARC patients had a median trough concentration of 7.70 µg/mL, and more than 60% had trough concentrations <10 µg/mL. In Chen et al. [31], median trough concentrations were 6.45 mg/L in ARC versus 10.72 mg/L in non-ARC patients (p < 0.001). In Sahraei et al. [23], AUC/MIC > 400 mg·h/L at MIC 1 mg/L was achieved in 82.14% with q8h dosing versus 46.42% with q12h dosing (p = 0.006), and trough concentrations > 15 µg/mL were achieved in 32.14% versus 0%, respectively (p < 0.001).

**Table 5:** Summary of Pharmacokinetic Findings PK, pharmacokinetic; ARC, augmented renal clearance; Ctrough, trough concentration; Cmax, maximum concentration; q6h/q8h/q12h, every 6/8/12 hours; AUC/AUC24h, (24-hour) area under the concentration-time curve; CL, clearance; Vd, volume of distribution; Vc, central volume of distribution; Css, steady-state concentration; t½, elimination half-life; CrCl, creatinine clearance; CKD-EPI, Chronic Kidney Disease Epidemiology Collaboration; C/D, concentration-to-dose ratio; MIC, minimum inhibitory concentration; PTA, probability of target attainment; NONMEM, nonlinear mixed-effects modelling software; ELF, epithelial lining fluid; k: elimination rate constant; ASPECT-NP: Assessment of the Safety Profile and Efficacy of Ceftolozane/Tazobactam in Nosocomial Pneumonia. †Very small sample caution required. ††Potential data overlap; see Methods.

Author	PK Outcomes Measured	Key PK Findings
Chu et al. [18]	Vancomycin Ctrough	Median vancomycin trough concentration in the ARC group was 7.70 µg/mL; more than 60% of ARC patients had trough concentrations below 10 µg/mL, indicating lower exposure than in the non-ARC comparator group within this cohort.
Sahraei et al. [23]	Cmax, Ctrough, k, t½, CL, Vd, AUC	q8h dosing produced significantly higher trough concentrations and AUC than q12h regimen in ARC patients. AUC/MIC >400 achieved 82.14% (q8h) vs 46.42% (q12h). Increased dosing frequency compensated substantially for ARC-related clearance.
Shorr et al. [22]	Plasma and ELF antibiotic exposure	Population PK simulations supported high PTA for ceftolozane and tazobactam in ARC; meropenem pharmacokinetic sampling was not performed in ASPECT-NP and should not be summarized as a measured PK finding from this study.
Razzazzadeh et al. [24]	Serum peak/trough; CL, t½, ke	Six-hour prolonged infusion of meropenem was associated with higher pharmacodynamic target attainment than the shorter prolonged-infusion regimens evaluated in this ARC cohort.
Troisi et al. [26]	Meropenem Css, CL	Strong linear relationship between measured creatinine clearance and meropenem clearance demonstrated. Measured (timed urine) CrCl provided more accurate dosing guidance than estimated equations (Cockcroft-Gault, CKD-EPI) in this population.
Fratoni et al. [27]†	CL, central volume (Vc), protein binding	Mean imipenem/relebactam clearance is higher in ARC patients than historical controls. Population PK model simulations predicted that the standard 1.25 g q6h regimen may maintain adequate PTA despite elevated clearance.
Mikami et al. [28]	Vancomycin Ctrough, concentration/dose ratio (C/D)	ARC patients had lower concentration-to-dose ratios than non-ARC patients, consistent with greater vancomycin clearance.
Alsultan et al. [29]	Meropenem CL, Vd	Mean meropenem clearance was 6.4 L/h and trough concentrations were highly variable; failure of PK/PD target attainment under standard dosing was reported in ARC patients.
Tang et al. [30]	Vancomycin Ctrough, CL, AUC24h, Vd	The ARC group had significantly higher clearance and substantially lower trough concentrations and AUC24h than non-ARC counterparts in this exclusively obstetric ICU cohort.
Nicolau et al. [17]†	AUC, Cmax, CL, t½, Vd (C/T)	ARC patients showed increased ceftolozane/tazobactam clearance and larger volume of distribution compared with healthy subjects.
He et al. [25]	Vancomycin Ctrough, AUC24h, CL, Vd	ARC patients exhibited higher vancomycin clearance and significantly lower trough concentrations than non-ARC patients. A higher weight-adjusted dose was required in ARC patients to achieve comparable exposure.
Chen et al. [31]	Vancomycin Ctrough	ARC patients had lower trough concentrations than patients with normal renal function (median: 6.45 mg/L versus 10.72 mg/L). Clinical prognosis was reported separately.
Chu et al. [19]††	Population PK parameters (CL, Vd)	A population PK model was developed in 95 ARC patients using NONMEM. Age was identified as an influential covariate on vancomycin clearance.

For β-lactam antibiotics, reduced exposure with higher renal function was again a recurring concern. In Troisi et al. [26], 46 critically ill adults undergoing continuous-infusion meropenem had significantly different renal function estimates when measured versus estimated methods were compared (measured CrCl: 74.7 mL/min versus CKD-EPI 103.1, Cockcroft-Gault 112.6, and Modification of Diet in Renal Disease (MDRD) 108.5 mL/min/1.73 m²; p < 0.001); these findings primarily highlight the importance of accurate renal function measurement for dose selection rather than providing direct evidence from an ARC-only cohort. In Shorr et al. [22], population PK simulations supported a high probability of target attainment for ceftolozane and tazobactam in 209 ARC participants. In the population PK study by Chu et al. [19], vancomycin concentrations from 95 ARC patients were analyzed with a nonlinear mixed-effects model in nonlinear mixed-effects modelling software (NONMEM), and age was retained as the most influential covariate on vancomycin clearance, supporting its inclusion in ARC-specific dosing models.

Pharmacodynamic and Clinical Outcomes

Table 6 shows that reduced PK/PD target attainment was common. Five studies reported at least one clinical or safety endpoint; however, only one study reported microbiologic cure/eradication, and two additional studies reported isolate MIC data without eradication outcomes. For meropenem, Razzazzadeh et al. [24] reported 50% fT > MIC attainment in 100% of patients receiving 1 g q8h over 6 h versus 40% with 2 g q8h over 3 h and 13% with 1 g q8h over 3 h (p = 0.0001); mean fT > MIC was 78.77±5.87%, 49.6±7.38%, and 43.2±7.98%, respectively (p = 0.0001). Clinical outcome data were otherwise limited and heterogeneous. In Shorr et al. [22], clinical cure in the ceftolozane/tazobactam arm was 59.4% in ARC versus 57.3% in normal renal function (difference: -2.1%, 95% CI: -14.8 to 10.8), mortality was 17.7% versus 17.6% (difference: 0.2%, 95% CI: -9.6 to 10.6), and microbiologic cure was 71.4% (55/77) versus 72.2% (70/97) (difference: 0.7%, 95% CI: -12.4 to 14.2). In Chen et al. [31], vancomycin troughs were lower in 26 ARC patients than in non-ARC patients (6.45 versus 10.72 mg/L; p < 0.001), whereas reported non-specific prognosis measures were not clearly worse in ARC. Across the review, five studies reported any clinical or safety endpoint; only one study reported a clinical cure, and only one study reported a microbiologic cure, which limited causal inference between PK non-attainment and patient-important outcomes.

**Table 6:** Pharmacodynamic and Clinical Outcomes PD, pharmacodynamic; PK/PD, pharmacokinetic/pharmacodynamic; ARC, augmented renal clearance; Ctrough, trough concentration; q6h/q8h/q12h, every 6/8/12 hours; AUC/AUC24h, (24-hour) area under the concentration-time curve; AUC/MIC, area under the concentration-time curve to minimum inhibitory concentration ratio; fT > MIC, free drug time above MIC; PTA, probability of target attainment; Css/MIC, steady-state concentration to MIC ratio; AKI, acute kidney injury; fAUC: area under the concentration-time curve of the unbound (free) drug; fT: fraction of the dosing interval/time; TDM, therapeutic drug monitoring; MIC, minimum inhibitory concentration; HABP, hospital-acquired bacterial pneumonia; VABP, ventilator-associated bacterial pneumonia; VAP, ventilator-associated pneumonia; MIPD, model-informed precision dosing. †Small sample limited precision. ††Potential data overlap.

Author	PD Outcomes	Clinical Outcomes	Microbiological Outcomes	Key Conclusions
Chu et al. [18]	Ctrough target attainment (10-20 µg/mL)	Not reported	Not reported	Most ARC patients in this cohort did not achieve the prespecified vancomycin trough target with standard dosing.
Sahraei et al. [23]	AUC/MIC > 400; Ctrough > 15 µg/mL	AKI incidence, mortality, length of stay	Not reported	q8h dosing was associated with higher PK/PD target attainment than q12h dosing, while reported mortality and AKI did not clearly differ between groups.
Shorr et al. [22]	PTA analysis (ceftolozane/tazobactam)	28-day all-cause mortality; clinical cure	Microbiological cure at test-of-cure	In the ARC subgroup, standard-dose ceftolozane/tazobactam was associated with a 59.4% clinical cure rate and 71.4% microbiologic cure at test-of-cure in HABP/VABP. Interpretation is limited by the post hoc subgroup design.
Razzazzadeh et al. [24]	fT > MIC target attainment	Clinical improvement parameters	Not reported; isolate MIC data only (not a microbiologic cure/eradication outcome)	Six-hour prolonged meropenem infusion was associated with higher fT > MIC attainment than the shorter infusion strategies studied in ARC patients with VAP.
Troisi et al. [26]	Css/MIC ratio	Not reported	Not reported	Measured urine creatinine clearance showed closer agreement with meropenem exposure targets than estimated equations in this continuous-infusion cohort.
Fratoni et al. [27]†	Joint PTA (imipenem fT > MIC; relebactam fAUC/MIC)	Not reported	Not reported	Model-based simulations predicted adequate PTA with standard dosing despite elevated clearance; this was based on a very small single-dose ARC cohort.
Mikami et al. [28]	Not formally reported	Not reported	Not reported	ARC was associated with lower vancomycin exposure, reflected by reduced concentration-to-dose ratios.
Alsultan et al. [29]	PK/PD target attainment	Not reported	Not reported	ARC was associated with reported failure of meropenem PK/PD target attainment under standard dosing.
Tang et al. [30]	Ctrough and AUC24h targets	Not reported	Not reported	ARC was associated with lower vancomycin exposure and reduced attainment of the reported PK/PD targets in the 41-patient vancomycin validation subgroup.
Nicolau et al. [17]†	fT > MIC (ceftolozane); threshold AUC (tazobactam)	Safety/tolerability outcomes only	Not reported	Adequate pharmacodynamic exposure was reported after a single 3 g ceftolozane/tazobactam dose in this phase 1 ARC study.
He et al. [25]	AUC24h/MIC calculations	Not reported	Not reported; MIC values used for AUC24/MIC calculations only	Higher vancomycin doses were required in ARC patients to achieve target AUC exposure.
Chen et al. [31]	Not formally reported	Length of stay and non-specific prognosis measures	Not reported	ARC patients had lower vancomycin trough attainment but similar reported clinical prognosis compared with non-ARC patients.
Chu et al. [19]††	Not reported	Not reported	Not reported	The population PK model was intended to support individualized ARC-adjusted dosing strategies using MIPD; this study was retained qualitatively and not included in pooled participant counts for explicit vancomycin target-attainment outcomes.

For vancomycin, failure to achieve target trough concentrations or AUC/MIC targets was frequent under standard dosing. In Sahraei et al. [23], q8h dosing improved AUC/MIC > 400 mg·h/L attainment at MIC 1 mg/L (82.14% versus 46.42%; p = 0.006) and trough attainment > 15 µg/mL (32.14% versus 0%; p < 0.001), without a statistically significant difference in 7-day acute kidney injury (28.6% versus 10.7%; p = 0.089) or mortality (25.0% versus 32.1%; p = 0.384).

Certainty of Evidence (GRADE)

Table 7 summarizes the certainty of evidence across the prespecified outcome domains. The certainty of evidence was very low for all assessed outcomes, including vancomycin PK/PD target non-attainment (five studies; 428 ARC participants), β-lactam PK/PD target non-attainment (six studies; 303 ARC participants), clinical cure or treatment success (one study; 209 ARC participants in a post hoc subgroup analysis), all-cause mortality (two studies; 265 ARC participants), microbiologic cure/eradication (one study; 147 evaluable ARC participants), and adverse drug reactions (one study; 56 ARC participants).

**Table 7:** Certainty of Evidence Across the Prespecified Outcome Domains GRADE certainty symbols: High = ⊕⊕⊕⊕; Moderate = ⊕⊕⊕⊖; Low = ⊕⊕⊖⊖; Very low = ⊕⊖⊖⊖. Downgrade reasons: -1 = serious concern; -2 = very serious concern. Starting certainty: randomized trials = High; observational studies = Low. ARC, augmented renal clearance; AUC, area under the concentration-time curve; AUC/MIC, AUC to minimum inhibitory concentration ratio; q6h/q8h/q12h, every 6/8/12 hours; AUC/AUC24h, (24-hour) area under the concentration-time curve; C/T, ceftolozane/tazobactam; CrCl, creatinine clearance; fT > MIC, free drug time above MIC; Css, steady-state concentration; GRADE, Grading of Recommendations Assessment, Development and Evaluation; ICU, intensive care unit; ITT, intention-to-treat; mITT, microbiologic ITT; MIC, minimum inhibitory concentration; PK/PD, pharmacokinetic/pharmacodynamic; PTA, probability of target attainment; RCT, randomized controlled trial; RoB, risk of bias; fT: fraction of the dosing interval/time; ASPECT-NP: Assessment of the Safety Profile and Efficacy of Ceftolozane/Tazobactam in Nosocomial Pneumonia. †Tang et al. [30] (Row 1): n = 21 ARC represents only those patients in the vancomycin treatment subgroup (n = 41 total: 21 ARC + 20 non-ARC), not the full obstetric cohort (n = 427; ARC n = 201). All clinical and PK/PD findings derive from a post hoc ARC subgroup analysis of the ASPECT-NP phase 3 RCT. PTA in Row 2 is simulation-derived (Monte Carlo); clinical outcome data (Rows 3-5) are from the ITT and mITT populations, respectively. Mortality data (Row 4) were not a prespecified primary or secondary outcome and were reported incidentally. AKI (Row 6) was a secondary outcome.

Outcome	Studies (n)	Participants (n)	Risk of Bias	Inconsistency	Indirectness	Imprecision	Publication Bias	Effect Summary	Certainty of Evidence (GRADE)
Vancomycin PK/PD target non-attainment (explicit trough or AUC/MIC target data)	5	428 ARC participants (Chu et al. [18] 186; Sahraei et al. [23] 56; He et al. [25] 139; Chen et al. [31] 26; Tang et al. [30] 21 † Tang et al. [30] contributes n = 21 ARC from the vancomycin validation subgroup only, not the 201 ARC in the full obstetric cohort.	Serious (-1)	Not serious (0)	Serious (-1)	Serious (-1)	Not formally assessable	Across five studies reporting explicit vancomycin target-attainment data, target non-attainment was common under standard dosing in ARC patients. Chu et al. [18]: >60% of ARC patients had trough concentrations <10 µg/mL. He et al. [25]: Subtherapeutic troughs (<10 mg/L) in 77.7% of ARC vs. 68.8% of non-ARC patients. Chen et al. [31]: Trough achievement rate 19.23% in ARC vs. 41.03% in non-ARC. Tang et al. [30]: 81.0% of ARC patients had trough <10 mg/L; 85.7% had AUC24h <400 mg·h/L. Sahraei et al. [23]: q8h dosing achieved AUC/MIC > 400 mg·h/L at MIC 1 mg/L in 82.14% of ARC patients vs. 46.42% with q12h dosing (p = 0.006).	⊕⊖⊖⊖ VERY LOW Predominantly small, heterogeneous observational studies reporting surrogate PK/PD outcomes. Downgraded: serious risk of bias (-1), serious indirectness (-1), serious imprecision (-1).
β-Lactam PK/PD target non-attainment (fT > MIC or PTA; by dosing strategy)	6	303 ARC participants (Razzazzadeh et al. [24] 45; Troisi et al. [26] 12; Alsultan et al. [29] 18; Shorr et al. [22] 209; Nicolau et al. [17] 11; Fratoni et al. [27] 8)	Serious (-1)	Serious (-1)	Serious (-1)	Serious (-1)	Not formally assessable	Findings were directionally heterogeneous across the six included studies: Studies showing reduced target attainment under standard dosing: Razzazzadeh et al. [24]: fT > MIC > 50% achieved in only 13% (group 1) and 40% (group 2) of ARC patients with standard infusion strategies; Alsultan et al. [29]: only 22% of ARC patients achieved the therapeutic PK/PD target vs. 64% of non-ARC patients (p = 0.016); Troisi et al. [26]: Css/MIC targets were affected by discrepancies between measured and estimated CrCl. Studies showing adequate or predicted-adequate PTA: Shorr et al. [22]: Monte Carlo simulation demonstrated >99% PTA for ceftolozane and >80% for tazobactam in ARC patients receiving C/T 3 g q8h; Fratoni et al. [27]: model-based simulations predicted adequate joint PTA for imipenem/relebactam with standard dosing despite elevated clearance in 8 ARC patients; Nicolau et al. [17]: adequate pharmacodynamic exposure was reported after single-dose ceftolozane/tazobactam in 11 ARC patients. Prolonged or continuous β-lactam infusion strategies improved fT > MIC attainment in studies that demonstrated PK/PD deficits. Clinical benefit of any dosing strategy remained uncertain across all studies.	⊕⊖⊖⊖ VERY LOW Predominantly observational and simulation-based PK evidence; directional heterogeneity across studies. Downgraded: serious risk of bias (-1), serious inconsistency (-1)
Clinical cure/treatment success	1	ARC subgroup of ASPECT-NP ITT (total ITT ARC n = 209) C/T arm: ARC n = 96; meropenem arm: ARC n = 113. Note: n = 209 represents two separate treatment arms from a comparative RCT, not a single therapeutic cohort. Clinical cure assessed per treatment arm.	Serious (-1)	Not applicable (0)	Serious (-1) (post hoc ARC subgroup analysis of parent RCT)	Serious (-1)	Not formally assessable	Shorr et al. [22]: post hoc ARC subgroup of ASPECT-NP. Clinical cure rates at test-of-cure were comparable between ARC and normal renal function within each treatment arm: C/T arm: ARC 59.4% vs. normal renal function 57.3% (ITT); treatment difference -2.1 (95% CI: -14.8 to 10.8). Meropenem arm: ARC 57.5% vs. normal renal function 59.3% (ITT); treatment difference 1.8 (95% CI: -10.6 to 14.2). The ARC subgroup analysis was not prespecified or powered for definitive clinical inference. No other included study reported clinical cure as an outcome.	⊕⊖⊖⊖ VERY LOW Single post hoc subgroup analysis from one RCT; results not powered for ARC-specific inference. Downgraded: serious risk of bias (-1), serious indirectness (-1), and serious imprecision (-1). Inconsistency was not assessable because only one study contributed to this outcome.
All-cause mortality	2	265 ARC participants across 2 studies (Shorr et al. [22] 209 ITT ARC; Sahraei et al. [23] 56 ARC) † Mortality data from Sahraei et al. [23] were reported incidentally in the Results section and were not a prespecified primary or secondary outcome of that trial.	Serious (-1)	Serious (-1) (only 2 studies; one with non-prespecified mortality data)	Serious (-1) (post hoc ARC subgroup; non-prespecified outcome in one study)	Very serious (-2)	Not formally assessable	Shorr et al. [22]: 28-day all-cause mortality was comparable between ARC and normal renal function within each treatment arm. C/T arm: ARC 17.7% vs. normal renal function 17.6% (treatment difference: 0.2 (95% CI: -9.6 to 10.6)). Meropenem arm: ARC 17.7% vs. normal renal function 20.3% (treatment difference: -2.6 (95% CI: -12.6 to 7.5)). Sahraei et al. [23]: Mortality 32.1% (9/28) in the q12h group vs. 25.0% (7/28) in the q8h group (p = 0.384). These data were not a prespecified outcome and should be interpreted with additional caution. Neither study demonstrated a statistically significant mortality difference. Inference is limited by small numbers, indirect subgroup design, and the non-prespecified nature of mortality reporting in one study.	⊕⊖⊖⊖ VERY LOW Only 2 studies; mortality not prespecified in one. Downgraded: serious risk of bias (-1), serious inconsistency (-1), serious indirectness (-1), very serious imprecision (-2).
Microbiologic cure/eradication	1	147 evaluable ARC participants (mITT population) C/T arm: n = 77 ARC; meropenem arm: n = 70 ARC Note: 147 = 77+70 across two separate treatment arms from a comparative RCT, not a single therapeutic cohort.	Serious (-1)	Not applicable (0)	Serious (-1) (post hoc ARC subgroup)	Very serious (-2)	Not formally assessable	Shorr et al. [22]: post hoc ARC subgroup of ASPECT-NP (mITT population). Per-participant microbiologic cure rates at test-of-cure were comparable across renal function groups within each treatment arm: C/T arm: ARC 71.4% (55/77) vs. normal renal function 72.2% (70/97); treatment difference: 0.7 (95% CI: -12.4 to 14.2). Meropenem arm: ARC 70.0% (49/70) vs. normal renal function 75.0% (66/88); treatment difference: 5.0 (95% CI: -8.7 to 19.0). No other included study reported microbiologic eradication, persistence, or relapse as an outcome. Inference is limited by the post hoc subgroup design and the absence of replication across independent studies.	⊕⊖⊖⊖ VERY LOW Single post hoc subgroup analysis; evaluable subgroup from one RCT. Downgraded for serious risk of bias (-1), serious indirectness (-1), and very serious imprecision (-2). Inconsistency was not assessable because only one study contributed to this outcome.
Adverse drug reactions (AKI/nephrotoxicity)	1	56 ARC participants (Sahraei et al. [23] 21: 28 q12h + 28 q8h)	Serious (-1)	Not applicable (0)	Serious (-1)	Very serious (-2)	Not assessable	Sahraei et al. [23]: AKI (defined as serum creatinine increase >0.3 mg/dL over 48h or anuria) at 7-day follow-up occurred in 3/28 (10.7%) patients in the q12h group and 8/28 (28.6%) in the q8h group (p = 0.089). No statistically significant difference was detected between dosing regimens, but precision was very limited given the small sample size. No other included study reported nephrotoxicity or adverse drug reactions as a formal outcome. AKI was a secondary outcome in this trial.	⊕⊖⊖⊖ VERY LOW Single small RCT. Downgraded: serious risk of bias (-1), serious indirectness (-1), very serious imprecision (-2).

Discussion

Principal Findings

This systematic review collated pharmacokinetic, pharmacodynamic, and limited clinical evidence from 13 studies published between 2020 and 2026 investigating the effect of ARC on vancomycin and β-lactam antibiotics in adults. The primary finding was a directional association between ARC, higher drug clearance, lower systemic exposure, and poorer PK/PD target attainment under standard dosing regimens. However, certainty of evidence remained very low because most included studies were observational, many outcomes were pharmacokinetic surrogates, and precision was limited by small or heterogeneous study populations. The confidence in individual results was also subject to risk of bias, in that more caution was taken with evidence that had confounding, missing data, or other problems. The second key finding was the paucity of patient-important clinical data. Five studies reported any clinical or safety endpoint, but only one study reported clinical cure, and only one study reported microbiologic cure. This disconnect between consistent pharmacokinetic findings and sparse clinical outcome evidence remains the main evidence gap in the field and limits the strength of any practice recommendations.

Comparison With Prior Literature

The pharmacokinetic data from the current review are generally supportive of prior data related to the effects of ARC on the pharmacokinetics of antimicrobial agents. ARC is known to affect the pharmacokinetics of hydrophilic, renally cleared antibiotics such as vancomycin and the β-lactams, as described by Hobbs et al. [5]. The background literature referenced in this section is not within the predetermined search period of 2020-2026 and has been provided only for the purposes of context; this background literature did not influence the identification, extraction, and analysis of data or risk of bias and was not considered in the systematic synthesis of findings. Specifically, for vancomycin, prior literature reviewed by Tesfamariam et al. [14] has described an association between increasing creatinine clearance and greater vancomycin clearance in ARC populations while also suggesting that conventional dose-intensification strategies may be insufficient to overcome ARC-associated hyperclearance. The vancomycin studies included in this review (Tables 4-6) are consistent with these observations, with median trough concentrations in ARC patients ranging from 6.45 to 7.70 µg/mL, substantially below the generally accepted target threshold of ≥10 µg/mL. While the directional finding of increased vancomycin clearance in ARC is consistent with Tesfamariam et al. [14], the present review differs in three meaningful respects. First, it incorporates β-lactam antibiotics alongside vancomycin, thereby capturing a broader and clinically relevant evidence base. Second, it applies the GRADE framework to each prespecified outcome domain, providing an explicit and reproducible certainty-of-evidence assessment that was absent from prior reviews in this area. Third, it treats variability in ARC definition and renal function ascertainment method not merely as a background consideration but as a primary methodological variable with direct consequences for cross-study comparability, an issue that becomes particularly apparent when timed urine creatinine clearance and serum-based estimation methods are used interchangeably across studies. Several included studies reported younger ARC cohorts, and some also suggested lower illness severity in ARC groups; this pattern is consistent with the physiological basis of ARC, namely a hyperdynamic circulatory state with augmented renal perfusion that may be more readily sustained in patients with preserved cardiovascular reserve. Body size, obesity, extracorporeal therapies, and concomitant nephroactive or dose-altering co-therapies may further contribute to between-patient variability in antibiotic exposure [32-33]. However, the contemporary literature reviewed systematically is not sufficient to prove an adverse effect on patient outcome by non-attainment of ARC-associated PK/PD targets, but previously published observational studies outside the scope of the review window period have shown that ARC was associated with reduced drug exposure and poor outcomes related to treatment in some ICU patients [15-16]. The previous studies are being mentioned here only as background evidence and have not been systematically analyzed. Chen et al. [31] reported significantly lower vancomycin troughs in ARC patients yet comparable non-specific prognosis measures, and Shorr et al. [22] observed similar clinical cure and mortality rates in ARC and non-ARC participants within treatment arms despite ARC-specific pharmacokinetic concerns. Several explanations are possible: ARC patients are often younger and less comorbid; conventional PK/PD thresholds may not translate directly into measurable short-term clinical differences in every ARC population; and the available studies were not powered or designed to test ARC-specific clinical hypotheses. Guideline-supported AUC-guided vancomycin dosing and TDM remain reasonable pharmacokinetic approaches [10-13], but direct evidence that these strategies improve clinical outcomes specifically in ARC remains limited. This disconnect highlights a central limitation of relying on pharmacokinetic surrogate endpoints alone and supports the need for prospective studies with prespecified clinical endpoints. Variability in the method used to determine ARC also affects more than simple classification. In critically ill patients, serum creatinine-based equations do not always reflect true renal function accurately, whereas measured urinary creatinine clearance may offer a more dependable guide for pharmacokinetic assessment and dose adjustment [34]. Tang et al. [30] used CKD-EPI eGFR, which may underestimate renal clearance in critically ill patients with dynamic physiology. Troisi et al. [26] showed that timed urine CrCl and serum creatinine-based equations yielded significantly different results in the same patients, with Cockcroft-Gault overestimating measured CrCl values by a substantial margin. These differences are clinically relevant because they affect ARC classification, dose selection, and the interpretation of exposure adequacy. Standardization of renal function ascertainment methods is therefore a prerequisite for interpretable evidence synthesis. Finally, the geographic concentration of included studies, with seven of 13 conducted at Chinese or Iranian institutions, may limit generalizability because vancomycin dosing norms, TDM intensity, MIC distributions, and local resistance patterns vary across settings.

Variability in Evidence and Evidence Quality

Among comparative observational studies, the principal sources of bias were confounding, participant selection, and incomplete outcome data. He et al. [25] remained of particular concern because participant selection was insufficiently described, and the study contributed to one of the larger vancomycin ARC cohorts. For randomized evidence, the main concerns were deviations from intended interventions and selection of reported results, with Shorr et al. [22] requiring additional caution because the ARC findings were derived from a post hoc subgroup analysis of a parent randomized trial. Non-comparative PK, modeling, and methodological studies were interpreted primarily through narrative appraisal rather than formal causal ROBINS-I inference. Despite consistent pharmacokinetic direction of effect, substantial variability remained in the magnitude of exposure reduction across studies, likely reflecting differences in dosing strategies, patient demographics, infusion methods, severity of illness, and renal function ascertainment.

Two studies included in this review had extremely small sample sizes: Fratoni et al. [27] (n = 8) and Nicolau et al. [17] (n = 11). Although these phase 1 and single-center PK studies provide mechanistic data on drug clearance in ARC, their estimates are imprecise and should not be used as stand-alone bases for clinical dosing recommendations. Their findings are best interpreted as hypothesis-generating and warrant confirmation in larger prospective cohorts.

The study by Alsultan et al. [29] was assessed to have a serious risk of bias due to confounding, as factors that could influence exposure and attainment of meropenem’s PK/PD target, such as variation in disease severity, renal function, and other clinical characteristics among patients, were not well controlled for in the comparison. Thus, the association found between ARC and reduced target attainment with meropenem should not be attributed solely to the latter. A study by Tang et al. [30] had concerns related to incomplete outcome data, as vancomycin exposure and target-attainment information were obtained for a subsample of all eligible individuals in the study. Thus, the validity of the results was questionable, as it was uncertain whether the selected sample was representative of the entire population of interest.

Clinical Implications

The predominant pharmacokinetic evidence across included studies (very low certainty, GRADE) suggests several pharmacokinetically plausible management considerations that may warrant individualized evaluation in practice, particularly in patients with confirmed ARC. These include earlier vancomycin TDM, more frequent vancomycin maintenance dosing when monitored exposure remains subtherapeutic, and prolonged or continuous infusion strategies for selected β-lactams. Of note, none of the studies included in this review evaluated TDM-guided dose adjustment as a prospectively defined intervention with prespecified clinical outcome endpoints in patients with ARC. The recommendation for AUC-guided vancomycin monitoring in this population therefore represents an extrapolation from general critical care and infectious disease guidelines [10-13] rather than a conclusion derived from ARC-specific interventional evidence. Whether systematic application of TDM, Bayesian dose optimization, or model-informed precision dosing in patients with confirmed ARC translates into measurable improvements in treatment success, mortality, or nephrotoxicity rates remains an unanswered clinical question.

All of these considerations are supported primarily by pharmacokinetic evidence and should not be interpreted as recommendations with established clinical outcome benefit. The current literature is insufficient to determine whether pharmacokinetic optimization strategies in ARC improve treatment success, mortality, microbiologic outcomes, or adverse events.

Limitations

The following limitations should be taken into account while interpreting this systematic review. It has been confined to the databases of PubMed, Scopus, and Cochrane Library without the inclusion of Embase, Web of Science, trial registries, and grey literature. The exclusion of Embase could have caused the absence of pharmacology-specific publications, conference abstracts, and unique indexed records. The limitation to the use of English-language publications released between 2020 and now has introduced the risk of language and time-window biases and implies the incompleteness of historical evidence on the antimicrobial exposure in patients with ARC. Moreover, the database-specific terminologies, PK/PD principles, and indexed filters used in search have potentially lowered search sensitivity. There were two vancomycin trials from one institution where the recruitment periods overlapped, so there is a possibility of participants' overlap. Most trials included PK/PD surrogate outcomes, while evidence regarding clinical cure, microbiologic cure, mortality, and adverse events was limited. Confounding, participant selection, missing data, and post hoc analysis increased the risk of bias.

Future Research Directions

Future investigations should focus on conducting multicenter prospective investigations as well as well-powered randomized trials in which a clear ARC population is included. Investigators must use a standardized definition of ARC and report whether the level of renal function was measured by means of measured or estimated creatinine clearance for improved comparability across studies. Pharmacokinetic investigations involving therapeutic drug monitoring, Bayesian dose adjustment, population pharmacokinetics, and pharmacodynamic models for precision dosing must be conducted prospectively using standardized protocols for dosing. Future randomized trials must go beyond evaluating pharmacokinetic markers of efficacy and should focus on clinically meaningful outcomes such as clinical cure, microbiological cure, mortality, length of stay, nephrotoxicity, and antimicrobial resistance. Comparative trials between standard dosing regimens and ARC-based intensification of dose or extended infusions of β-lactams are especially important.

## Conclusions

In the contemporary studies evaluated, the main pharmacokinetic pattern seen in association with increased renal clearance has been high vancomycin and β-lactam clearance, low systemic exposure, and low pharmacokinetic/pharmacodynamic target attainment under standard dosing regimens; however, there is very low certainty in these observations. Pharmacokinetically reasonable interventions to optimize target attainment include therapeutic drug monitoring-based dosing adjustment, frequent vancomycin maintenance dosing, and prolonged or continuous β-lactam infusions; however, evidence from existing studies does not confirm an improvement in patient-specific important outcomes in patients with ARC from such an approach. The above conclusions were reached based on the contemporary literature evaluated in the accessible literature search, and they should not be considered as covering the full history of the evidence on antimicrobial treatment of patients with ARC. Large prospective multicenter studies are required to define if ARC-oriented antimicrobial dosing improves outcomes in ARC.

## Appendices

Appendix 1 

**Table 8:** Database Search Strings

Database	Search Strategy	Filters/Limits
PubMed	("Augmented Renal Clearance" [Title/Abstract] OR "increased renal clearance" [Title/Abstract] OR "enhanced renal clearance" [Title/Abstract] OR ARC[Title/Abstract] OR ("Glomerular Filtration Rate" [MeSH] AND (augmented[Title/Abstract] OR increased[Title/Abstract]))) AND ("beta-Lactams"[MeSH] OR beta-lactam*[Title/Abstract] OR penicillin*[Title/Abstract] OR cephalosporin*[Title/Abstract] OR carbapenem*[Title/Abstract] OR piperacillin[Title/Abstract] OR meropenem [Title/Abstract] OR cefepime[Title/Abstract] OR "Vancomycin"[MeSH] OR vancomycin[Title/Abstract]) AND ("Drug Dosage"[MeSH] OR "Pharmacokinetics"[MeSH] OR "dose adjustment"[Title/Abstract] OR "extended infusion"[Title/Abstract] OR "continuous infusion"[Title/Abstract] OR "PK/PD"[Title/Abstract]) AND (adult[MeSH]) NOT (review[Publication Type] OR case reports[Publication Type])	English language; Humans; Adults (≥18 years); January 1, 2020, to January 31, 2026
Cochrane Library	("renal clearance" OR "high clearance" OR "increased clearance" OR "augmented clearance"):ti,ab,kw AND (vancomycin OR "beta-lactam*" OR penicillin* OR cephalosporin* OR carbapenem* OR piperacillin OR tazobactam OR meropenem OR imipenem OR cefepime OR ceftriaxone):ti,ab,kw AND (dosing OR "dose adjust*" OR "extended infusion" OR "continuous infusion" OR pharmacokinetic* OR "PK/PD" OR TDM OR AUC):ti,ab,kw	Limited to trials and studies indexed in Cochrane Library; 2020-2026
Scopus	TITLE-ABS-KEY ("augmented renal clearance" OR "increased renal clearance" OR "high renal clearance" OR ARC OR ("creatinine clearance" AND (augmented OR increased OR high))) AND TITLE-ABS-KEY (vancomycin OR "beta-lactam*" OR penicillin* OR cephalosporin* OR carbapenem* OR piperacillin OR tazobactam OR meropenem OR imipenem OR cefepime OR ceftolozane) AND TITLE-ABS-KEY (dosing OR "dose adjust*" OR "extended infusion" OR "prolonged infusion" OR "continuous infusion" OR pharmacokinetic* OR "PK/PD" OR TDM OR AUC)	English language; Human studies; 2020-2026
